# Efficacy and safety of belimumab for the treatment of refractory childhood-onset systemic lupus erythematosus: A single-center, real-world, retrospective study

**DOI:** 10.3389/fimmu.2022.1067721

**Published:** 2022-12-14

**Authors:** Dahai Wang, Chunrong Shan, Jia Liu, Ranran Zhang, Guohao Zhu, Tingting Gao, Hong Chang, Shan Gao, Cui Bai, Nana Nie, Qiuye Zhang, Yi Lin

**Affiliations:** ^1^ Department of Nephrology, Rheumatology, and Immunology, The Affiliated Hospital of Qingdao University, Qingdao, China; ^2^ Department of Pediatrics, Qingdao Women and Children’s Hospital, Qingdao, China

**Keywords:** systemic lupus erythematosus (SLE), childhood-onset SLE (cSLE), belimumab, treatment, safety

## Abstract

**Objective:**

This study aimed to investigate the efficacy and safety of belimumab for treating children with refractory childhood-onset systemic lupus erythematosus (cSLE).

**Methods:**

Twenty-six cSLE patients who received belimumab treatment in our hospital from January 2020 to September 2021 (23 of them for more than 52 weeks) were enrolled in this study. Their clinical and laboratory data, assessment of disease activity, glucocorticoid dosage, and treatment-emergent adverse events (TEAEs) were retrieved for analysis. The paired samples *t-*test and the nonparametric test were used to compare the baseline and post-treatment data.

**Results:**

The mean age of onset was 10.3 ± 2.4 years old; the mean disease duration was 41.6 ± 37.4 months; the median Systemic Lupus Erythematosus Disease Activity Index 2000 (SLEDAI-2K) score was 10 (*P*_25_, *P*_75_: 3, 17); and the mean Physician’s Global Assessment (PGA) score at baseline was 1.9 ± 1.0. Compared with the baseline values, there was a significant decrease in the 24-h urine protein quantifications at 24 and 52 weeks of treatment (*P*<0.05) as well as an elevated complement (C) 3 and C4 levels at 4, 12, 24, and 52 weeks of treatment. In addition, the SLEDAI-2K and PGA scores as well as the percentage of CD19^+^ B cells were significantly decreased at 12, 24, and 52 weeks of treatment compared with the baseline values (*P*<0.05). The dosage of glucocorticoid at 4, 12, 24, and 52 weeks of treatment was significantly less than that at baseline or the previous follow-up (*P*<0.05). At 52 weeks, 14 subjects (53.8%) achieved Lupus Low Disease Activity State (LLDAS), and 4 subjects (15.4%) reached clinical remission (CR). At the last follow-up, 16 subjects (61.5%) achieved LLDAS, and 10 subjects (38.5%) reached CR.

**Conclusions:**

Belimumab treatment can significantly improve laboratory indicators, reduce disease activity, and decrease the dosage of glucocorticoid required in children with cSLE. Moreover, it has a good safety profile.

## Introduction

Systemic lupus erythematosus (SLE), a systemic autoimmune disease, is characterized by the generation of autoantibodies, widespread inflammation, and tissue damage in multiple organs; it also presents with a high degree of clinical heterogeneity (1). In particular, systemic manifestations and organ involvement, including the kidney, central nervous system, blood, etc., and a worse prognosis (2, 3) are more common in childhood-onset SLE (cSLE) compared with SLE in adults.

The current therapeutic approaches for SLE involve glucocorticoids (hormones), immunosuppressants, antimalarials, and biologics (4), among which belimumab is the only biologic that has been approved by both the National Medical Products Administration (NMPA) of China and the Food and Drug Administration (FDA) of the USA. In addition, antimalarials (such as hydroxychloroquine) and glucocorticoids are still the most common drugs used for treating cSLE patients who can be treated with belimumab if their age is more than 5 years old and if they are experiencing poor disease control with conventional treatment (5).

Belimumab is a recombinant fully human monoclonal antibody that can specifically recognize and inhibit the biological activity of B-lymphocyte stimulator (BLyS) by binding to and neutralizing soluble BLyS, leading to inhibition of autoreactive B cell survival, promotion of apoptosis in autoreactive B cells, and reduction of autoreactive B cell differentiation into autoantibody-producing plasma cells. However, belimumab treatment has less of an effect on advanced cells and preserves immunity (6–8).

Nevertheless, there are currently few studies on the clinical application of belimumab in pediatric populations (9), especially its treatment for refractory cSLE. Therefore, the efficacy and safety of belimumab for the treatment of cSLE patients need to be verified. To this end, our study collected the real-world clinical data of cSLE patients who were treated with belimumab in a single center; these data were used to analyze the efficacy and safety of belimumab in this population.

## Materials and methods

### Research subjects

A total of 26 patients with cSLE were included in this study, all of whom received belimumab treatment during the period from January 2020 to September 2021 in the Department of Pediatric Renal, Rheumatology, and Immunology of Qingdao University Affiliated Hospital. The main inclusion criteria of the participants in this study were as follows: 1) aged 5–18 years old; 2) diagnosed with SLE; 3) diagnosed with refractory SLE, which met at least one of the following criteria: a) Systemic Lupus Erythematosus Disease Activity Index 2000 (SLEDAI-2K) score was still greater than 6 after 3 months of standard therapy (including glucocorticoids, antimalarials, and immunosuppressants); b) failure to respond to standard therapy in any system or organ for more than 6 months; c) relapse in any system or organ after initial clinical remission; d) the prednisone dose could not be reduced to ≤5 mg after 1 year of standard treatment. Moreover, the main exclusion criteria of the subjects were as follows: 1) patients who were allergic to the active substance of belimumab or any excipients; 2) those who received fewer than three doses of belimumab; 3) those who had a history of treatment with biological agents, such as belimumab or rituximab. All patients received belimumab at a dosage of 10 mg/kg every 2 weeks for the first three doses, then every 4 weeks thereafter.

### Study design

This clinical study was a retrospective, single-center, case-series study. The diagnosis of SLE was evaluated based on the 2019 European League Against Rheumatism/American College of Rheumatology classification criteria for SLE (10).

Briefly, the Lupus Low Disease Activity State (LLDAS) (11) was considered under the following conditions: 1) SLEDAI-2K ≤4, with no activity in major organ systems (renal, central nervous system, cardiopulmonary) and no vasculitis, fever, hemolytic anemia, or gastrointestinal activity; 2) no new lupus disease activity compared with the previous assessment; 3) Physician’s Global Assessment (PGA) score ≤1; 4) a current prednisolone (or equivalent) dose ≤7.5 mg daily; 5) well-tolerated standard maintenance doses of immunosuppressive drugs and approved biological agents.

Moreover, clinical remission (CR) on therapy could be considered under the following conditions: PGA<0.5, SLEDAI-2K=0, with low-dose corticosteroids (prednisone ≤5 mg/day), and stable maintenance with antimalarials, immunosuppressives, and/or biologics (12).

### Data collection

The collected data included complete basic information about the patients, clinical data, SLE disease activity assessment and adverse reactions at baseline, and assessment of the efficacy of belimumab after treatment for 4 weeks, 12 weeks, 24 weeks, and 52 weeks. The basic information included the age of disease onset, sex, cause of medication, course of illness, etc. The clinical data included clinical manifestations of the affected organs, laboratory tests of the blood and urine, pathology of the renal biopsy, and oral hormone doses. The laboratory tests included the detection of anti-double-stranded DNA (anti-dsDNA) antibody (western blot analysis); measurement of the levels of complement (C) 3, C4, and the percentage of B cells (with the marker CD19^+^); and the 24-h urine protein quantification. SLE disease activity assessment included the SLEDAI-2K score and the PGA score. Finally, treatment-emergent adverse events (TEAEs) included infusion reactions, allergic reactions, infections, leukopenia, gastrointestinal disorders, psychiatric disorders, nervous system disorders, and musculoskeletal disorders.

### Informed consent

Our study was approved by the Medical Ethics Committee of the Affiliated Hospital of Qingdao University (File number: QYFYWZLL26705), and the legal guardians of the children all gave informed consent and signed the informed consent form.

### Statistical analysis

All data were analyzed with SPSS 25.0 statistical software. The data conforming to a normal distribution were represented by the mean ± standard deviation, and the comparison of the difference between the two groups was statistically analyzed using ANOVA of repeated measures. Meanwhile, the data that do not conform to a normal distribution were represented by the median (*P*_25_, *P*_75_), and the difference between the two groups (including hierarchical data) was compared using the nonparametric test. The count data were represented by a percentage (n [%]), and the difference between the two groups was compared using the chi-squared test or Fisher’s exact test. *P*<0.05 was considered a statistically significant difference between groups.

## Results

### Characteristics of the cSLE patients

A total of 26 patients with cSLE who received belimumab were enrolled in this study. The baseline demographic and clinical characteristics of the patients are indicated in **Table 1**. All recruited patients received eight or more doses (≥24 weeks) of belimumab, among which only three subjects did not complete 52 weeks of treatment, including one subject who stopped the belimumab treatment by herself, while the other two subjects used telitacicept instead of belimumab due to poor treatment effects.

**Table 1:** The baseline demographic and clinical characteristics of 26 childhood-onset systemic lupus erythematosus patients.

Characteristic	Belimumab group (n=26)
Age of onset, years	10.3 ± 2.4
Age at enrollment, years	13.7 ± 2.7
Sex	
Female	21/26 (80.8%)
Male	5/26 (19.2%)
Disease duration, months	41.6 ± 37.4
Follow-up time, months	19.6 ± 6.4
Times of belimumab therapy	
Five times (≥12 weeks)	26/26 (100%)
Eight times (≥24 weeks)	26/26 (100%)
Fourteen times (≥52 weeks)	23/26 (88.5%)
Organs involved	
Fever	6/26 (23.1%)
Skin	11/26 (42.3%)
Central nervous system	2/26 (7.7%)
Kidney	18/26 (69.2%)
Lung	1/26 (3.8%)
Blood system	12/26 (46.2%)
Cardiovascular	3/26 (11.5%)
Muscle and joint	4/26 (15.4%)
Digestive tract	1/26 (3.8%)
Renal biopsy	14/26 (53.8%)
Class I	1/14 (7.1%))
Class III	2/14 (14.3%)
Class IV	7/14 (50.0%)
Class V	2/14 (14.3%)
Class III+V	1/14 (7.1%)
Class IV+V	1/14 (7.1%)
Immunological indicators at enrollment	
ANA positive	25/26 (96.2%)
Anti-dsDNA antibody positive	17/26 (65.4%)
Complement 3 (reference range: 0.9–1.8), g/L	0.70 ± 0.40
Complement 4 (reference range: 0.1–0.4), g/L	0.14 ± 0.08
Disease evaluation at enrollment	
SLEDAI-2K score	10 (3, 17)
PGA score	1.9 ± 1.0
Combined drugs	
Glucocorticoid	26/26 (100%)
Antimalarial	25/26 (96.2%)
Immunosuppressants	
CYC only	5/26 (19.2%)
MMF after CYC	5/26 (19.2%)
MMF only	13/26 (50%)
MMF+FK	2/26 (7.7%)
FK only	1/26 (3.8%)

ANA, Antinuclear antibodies; SLEDAI, Systemic Lupus Erythematosus Disease Activity Index; PGA, Physician’s global assessment; CYC, Cyclophosphamide; MMF, Mycophenolate mofetil; FK, Tacrolimus.

### Changes in clinical manifestations before and after treatment with belimumab

**Figure 1:**
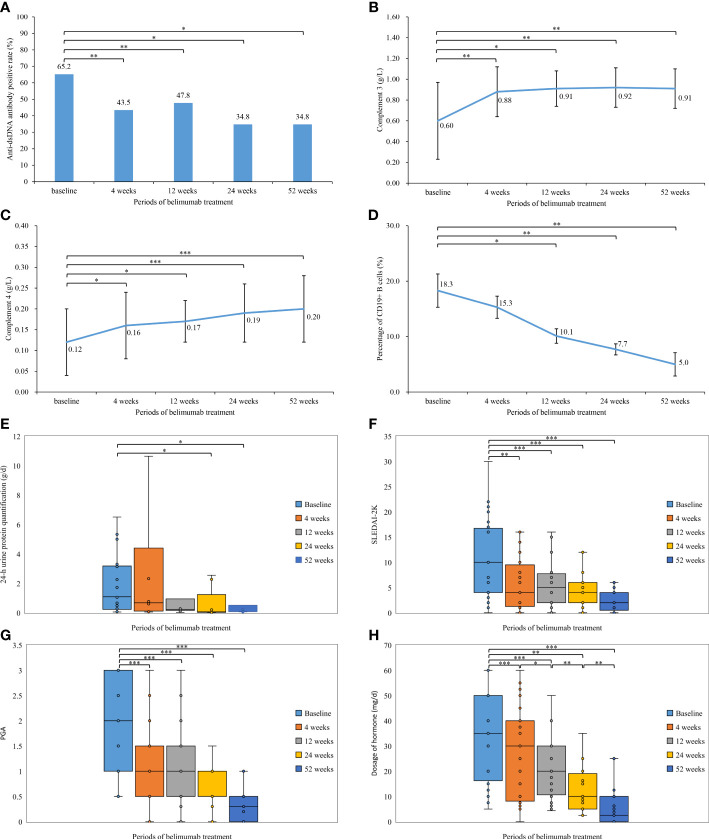
Comparison of the laboratory tests, disease activity, and hormone dosage during the different periods of belimumab treatment (up to 52 weeks) in 23 childhood-onset systemic lupus erythematosus patients. **(A)** Anti-dsDNA antibody positive rate (%). **(B)** Complement 3 (g/L). **(C)** Complement 4 (g/L). **(D)** Percentage of CD19^+^ B cells (%). **(E)** 24-h urine protein quantification (g/d). **(F)** Systemic Lupus Erythematosus Disease Activity Index 2000. **(G)** Physician’s Global Assessment. **(H)** Dosage of hormone (mg/d). *P< 0.05; **P< 0.01; ***P < 0.001.

In our study, 23 patients received belimumab treatment for more than 52 weeks, and the mean follow-up time was 20.5 ± 6.1 months. Changes in their clinical manifestations before and after treatment with belimumab are shown in **Table 2** and **Figure 1**. After 4 weeks, 12 weeks, 24 weeks, and 52 weeks of treatment, the positive rate of anti-dsDNA antibodies was significantly decreased compared to that at baseline (*P*<0.05). In addition, the levels of C3 and C4 after 4 weeks, 12 weeks, 24 weeks, and 52 weeks of treatment were significantly greater than those at baseline (*P*<0.05). There were no statistically significant differences in the 24-h urine protein quantification between the 4-week and 12-week treatment groups and the baseline group (*P*>0.05); however, the difference in this value between the longer treatment (for 24 and 52 weeks) and at baseline was statistically significant (*P*<0.05). Furthermore, there was no statistically significant change in the percentage of CD19^+^ B cells after 4 weeks of treatment compared to that at baseline (*P*>0.05); but after 12 weeks, 24 weeks, and 52 weeks of treatment, the percentage of CD19^+^ B cells was significantly decreased compared with that at baseline (*P*<0.05). Meanwhile, after 4 weeks, 12 weeks, 24 weeks, and 52 weeks of treatment, the SLEDAI-2K and PGA scores were significantly less than those at baseline (*P*<0.05). Similarly, the doses of corticosteroids were significantly reduced compared with those at baseline (*P*<0.05) as well as at the previous follow-up.

**Table 2:** Comparison of the laboratory tests, disease activity, and hormone dosage during the different periods of belimumab treatment (up to 52 weeks) in 23 childhood-onset systemic lupus erythematosus patients.

Characteristic		Baseline	4 weeks	12 weeks	24 weeks	52 weeks
Anti-dsDNA antibody	Positive rate (%)	65.2	43.5	47.8	34.8	34.8
	*P^a^*		0.003	0.001	0.019	0.019
Complement 3	g/L	0.60 ± 0.37	0.88 ± 0.24	0.91 ± 0.17	0.92 ± 0.19	0.91 ± 0.19
	*MD (95%CI)*		0.278 (0.074 to 0.483)	0.302 (0.054 to 0.550)	0.319 (0.102 to 0.537)	0.308 (0.093 to 0.522)
	*P*		0.004	0.011	0.002	0.002
Complement 4	g/L	0.12 ± 0.08	0.16 ± 0.08	0.17 ± 0.05	0.19 ± 0.07	0.20 ± 0.08
	*MD (95%CI)*		0.044 (0.008 to 0.080)	0.049 (0.005 to 0.093)	0.071 (0.030 to 0.112)	0.084 (0.035 to 0.133)
	*P*		0.010	0.022	< 0.001	< 0.001
24-h urine protein quantification	g/d	1.05 (0.12, 3. 14)	0.6 (0.07, 2.32)	0.2 (0.11, 0.32)	0.06 (0.03, 1.24)	0.1 (0.07, 0.9)
	*Z*		-0.681	-1.014	-2.521	-2.520
	*P*		0.496	0.309	0.012	0.012
Percentage of CD19^+^ B cells	%	18.3 ± 3.0	15.3 ± 2.0	10.1 ± 1.3	7.7 ± 1.0	5.0 ± 0.4
	*MD (95%CI)*		-3.011 (-8.266 to 2.244)	-8.218 (-15.162 to -1.274)	-10.626 (-18.190 to -3.063)	-13.356 (-22.429 to -4.282)
	*P*		0.878	0.013	0.002	0.001
SLEDAI-2K	score	10 (4, 17)	4 (1, 10)	6 (2, 8)	4 (2, 6)	2 (0, 4)
	*Z*		-3.416	-3.640	-3.726	-3.885
	*P*		0.001	< 0.001	< 0.001	< 0.001
PGA	score	2 (1, 3)	1 (0.5, 1.5)	1 (0.5, 1.5)	0.5 (0.5, 1)	0.3 (0, 0.5)
	*Z*		-3.960	-3.950	-3.938	-4.208
	*P*		<0.001	<0.001	< 0.001	< 0.001
Dosage of hormone	mg/d	40 (20, 50)	30 (10, 40)	20 (12.5, 30)	10 (5, 20)	2.5 (0, 10)
	*Z*		-3.764	-3.706	-3.266	-4.080
	*P*		<0.001	<0.001	0.001	<0.001
	*Z^a^*		-3.761	-2.340	-3.029	-3.420
	*P^b^*		<0.001	0.019	0.002	0.001

ANA, Antinuclear antibody; SLEDAI-2K, Systemic Lupus Erythematosus Disease Activity Index 2000; PGA, Physician’s Global Assessment; MD, Mean difference; CI, Confidence interval. Reference range: Complement 3: 0.9–1.8 g/L, Complement 4: 0.1–0.4 g/L, 24-h urine protein quantification: <0.15 g, Percentage of CD19^+^ B cells: 7.3–18.2%. The *P*-values are compared to the baseline values, the *P* ^a^ values are calculated by Fisher’s exact test, and the *Z* ^a^-values and *P* ^b^-values are compared with the values at the previous follow-up.

**Figure 2:**
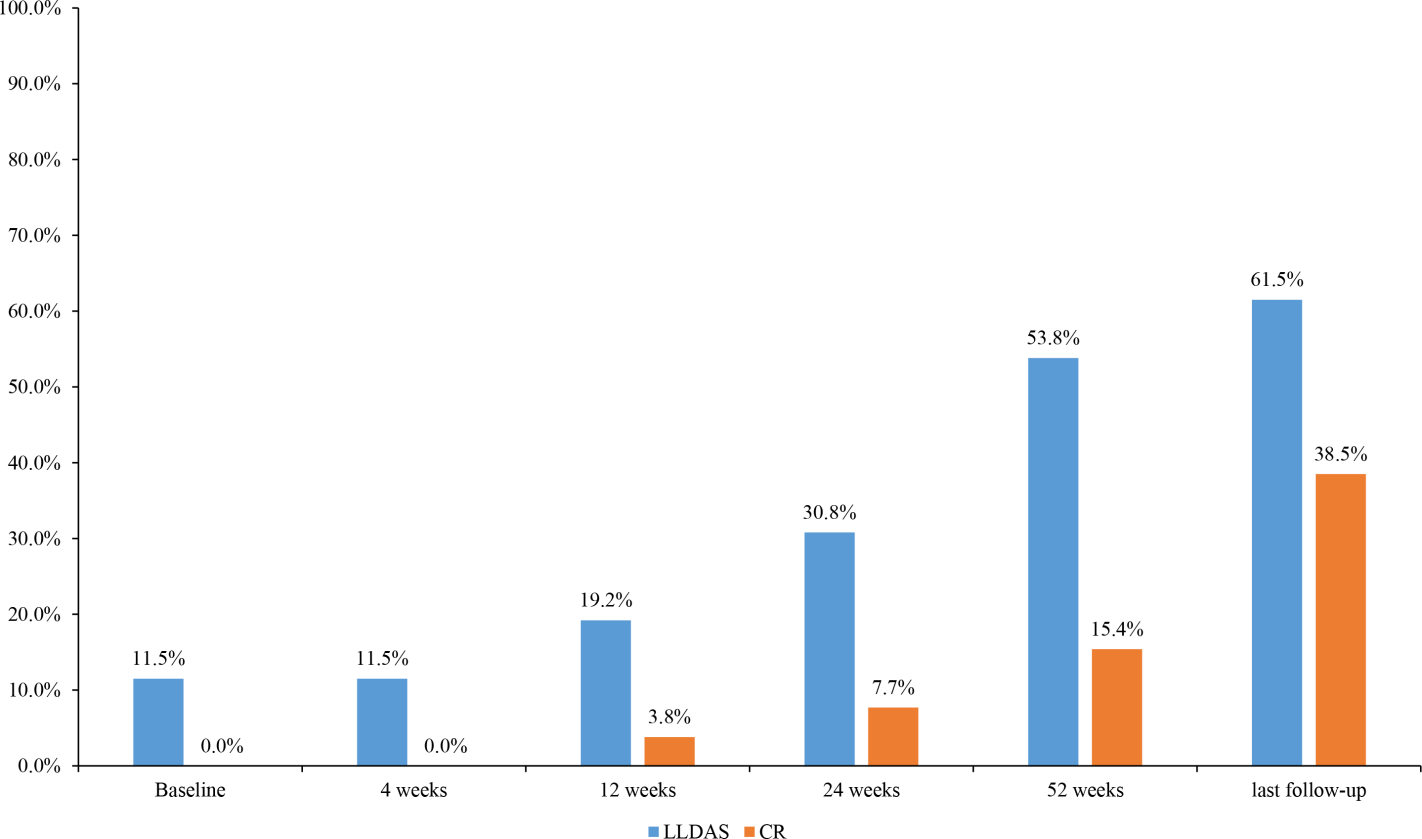
Changes in the proportion of disease activity states during the different periods of belimumab treatment in 26 childhood-onset systemic lupus erythematosus patients. LLDAS, Lupus Low Disease Activity State; CR, clinical remission.

Among the 26 patients who received belimumab (23 patients more than 52 weeks), 20 subjects (76.9%) had a PGA score of ≤1, and 21 subjects (80.8%) had a SLEDAI-2K score of ≤6 points at 52 weeks. In the evaluation of the therapeutic efficacy of belimumab on SLE, there were 14 patients (53.8%) who achieved LLDAS and 4 patients (15.4%) who achieved CR on therapy (**Figure 2**).

At the last follow-up, there were 11 subjects (42.3%, 11/26) who stopped taking corticosteroids, 17 subjects (65.4%) who reduced the dose of prednisone to ≤5 mg, 16 subjects (61.5%) who achieved LLDAS, and 10 subjects (38.5%) who achieved CR on therapy.

### TEAEs

During the period of treatment with belimumab, there were three patients with TEAEs, including one subject who suffered from fungal pneumonia, one subject who suffered from an upper respiratory tract infection and a gastrointestinal infection, and one patient who suffered from limb pain. All of these cases were classified as mild-moderate TEAEs, and all three patients with TEAEs responded well to symptomatic treatments (**Table 3**).

**Table 3:** Treatment-emergent adverse events (TEAEs) that occurred during the period of belimumab treatment.

TEAEs	Belimumab (n=26)
Number of patients with TEAEs, n (%)	3 (11.5%)
Infusion reaction	0
Allergic reaction	0
Infection	3
Upper respiratory tract infection	1^a^
Lower respiratory tract infection	1
Urinary tract infection	0
Herpes simplex	0
Herpes zoster	0
Gastrointestinal infection	1^b^
Leukopenia	0
Gastrointestinal disorders	0
Psychiatric disorders	0
Nervous system disorders	0
Musculoskeletal disorders	1

^a^ and ^b^ appeared in the same patient.

## Discussion

A variety of biological drugs have been recently developed for treating SLE patients. In particular, belimumab was approved for SLE in children by the FDA of the USA in April 2019 and the NMPA of China in December 2020. To date, belimumab is the only biologic agent approved to treat SLE patients older than 5 years old. The clinical application of belimumab in children has been increasing since its approval; however, reports on the clinical data of belimumab for the treatment of cSLE patients are limited. To this end, our study collected the clinical data of 26 children with cSLE who were treated with belimumab in a single center; these data were used to analyze the efficacy and safety of belimumab in this population.

In 26 cSLE patients, the longest duration of belimumab treatment was 2 years and 7 months. In China, both the sample size and the duration of medication use of this study make it a top-ranked study on cSLE. It has been reported that belimumab can be used for SLE patients with a poor response to conventional treatment or recurrent SLE (5, 13–16). At present, the definition of refractory SLE is not clear. In general, if patients do not respond to standard therapy or the disease relapses after initial clinical remission, those cases are considered refractory (17, 18). Based on the literature and our experience, we developed a definition of refractory SLE that was used for enrolling the refractory SLE patients in the study. In our study, the median course of the disease was 41 months, and the median SLEDAI-2K score of the cSLE patients indicated moderate activity at baseline. The main reasons for treatment with belimumab were disease progression, unable to control the SLE activity by the current regimen, and an inability to reduce the hormone dose to ≤5 mg, as previously reported (19, 20). The most common involved organs of the enrolled patients were the kidney (up to 69.2%), followed by the blood system, and the skin, similar to the previous report by the Chinese Systemic Lupus Erythematosus Treatment and Research Group (21). However, the proportion of renal involvement in our study was significantly greater than that reported previously (21), suggesting that lupus nephritis in children is more difficult to treat and more likely to develop refractory cases.

The Evaluation of Use of Belimumab in Clinical Practice Settings (OBSErve) study, a multinational cohort study program in the USA, demonstrated that the levels of C3 and C4 after 24 months of treatment with belimumab were significantly greater than those at baseline (22). While in a Chinese study of adults, the C3 and C4 levels were significantly elevated after an average of three months of belimumab treatment (23). In this study, through uninterrupted follow-up observation, we found that compared with the baseline levels, the levels of C3 and C4 were increased, but the positive rate of anti-dsDNA antibody was significantly decreased after 4 weeks of treatment with belimumab. In addition, the elevated C3 and C4 levels and reduced positive rates of anti-dsDNA antibodies were maintained during the period of subsequent treatment and follow-up compared to the baseline values, suggesting that belimumab may increase the C3 and C4 levels as early as the third dose.

Moreover, a multicenter, randomized, double-blind, placebo-controlled study has revealed that in adult patients with lupus nephritis, the rate of improvement in the ratio of urine protein creatinine was significantly increased after two years of treatment with belimumab (24), but another study has demonstrated no significant differences of the 24-h urinary protein quantification between at baseline and after belimumab treatment for various durations (6, 12, and 24 months) (22). Due to the limited number of clinical reports on the role of belimumab in the treatment of children with lupus nephritis, we performed this study. We found that the 24-h urinary protein level was significantly decreased after 24 and 52 weeks of belimumab treatment compared to the baseline level, suggesting that belimumab may be effective for treating children with lupus nephritis. However, treatment for at least six months may be needed to observe an effect.

BLyS is a soluble ligand of the tumor necrosis cytokine family and can induce B cell differentiation, homeostasis, and selection. Belimumab can bind to BLyS and further reduce the differentiation of B cells into plasma cells to reduce the production of autoantibodies. Therefore, the level of B lymphocytes in the peripheral blood after belimumab treatment can reflect its therapeutic efficacy. THU0231 Belimumab in Paediatric SLE (the PLUTO study) has shown that the level of total B cells was decreased in the belimumab group after 52 weeks of treatment compared to that in the placebo group (20). In this study, the percentage of CD19^+^ B lymphocytes decreased significantly after 12 weeks of belimumab treatment compared with that at baseline, supporting the significant inhibitory effect of belimumab on B lymphocytes. This effect was observed after treatment with the fourth dose.

The SLEDAI-2K and PGA scores are important indicators for evaluating SLE activity, and the treat-to-target (T2T) principle also has been well accepted. Moreover, LLDAS, CR on treatment, and CR off treatment are the three most important target states of SLE patients (25). LLDAS is a reachable goal of T2T for the treatment of cSLE. For example, in Wahadat’s study, the follow-up of 51 cSLE patients showed that all patients achieved LLDAS after 186 days of treatment, on average (26). Another study has demonstrated that belimumab reduced the disease activity as evaluated by the SLEDAI-2K score and the risk of recurrence (27). Additional examples of the clinical application of belimumab also have been reported recently and are mentioned below. Within one year of treatment with belimumab, the proportion of patients achieving low disease activity reached more than 70% (28). Moreover, a multi-center real-world study in China, in which our center participated, also showed that the proportions of SLE patients with LLDAS or CR after 28 weeks of belimumab treatment were 40.5% and 9%, respectively; while in the traditional drug treatment group, the proportions of SLE patients with LLDAS or CR were only 8.8% and 3.5%, respectively (29). It is notable that the data of our center which is an important part of the Chinese multi-center real-world study showed that the proportions of SLE patients with LLDAS or CR in the traditional drug treatment group were 16.7% and 8.3%. In our study, the SLEDAI-2K and PGA scores were significantly decreased after 4 weeks of belimumab treatment compared to those at baseline. In addition, 53.8% of the enrolled patients achieved LLDAS and 15.4% of the subjects reached CR on therapy within one year of treatment. Meanwhile, at the time of the last follow-up, 61.5% of the patients achieved LLDAS and 38.5% of the subjects reached CR on therapy. All of these results are consistent with those reported in the literature. In our study, some patients still had active SLE after belimumab treatment. Among the three patients who interrupted the treatment with belimumab, one subject stopped the belimumab treatment by herself due to economic reasons; another subject suffered from severe activity after self-discontinuation of treatment, including hemocytopenia, massive proteinuria, and chronic renal failure, but severe activity remained after treatments with methylprednisolone pulse, cyclophosphamide pulse, tacrolimus, and eight doses of belimumab. Therefore, she gave up belimumab, and instead took telitacicept. The third patient replaced belimumab with telitacicept due to continuous abnormal immunological indicators after eight doses of belimumab treatment. Thus, these three patients were excluded from the data analysis, which may lead to an overestimate of belimumab efficacy; this is a limitation of this retrospective study. Furthermore, the basic treatment was not able to reduce the dose of hormone to less than 5 mg in some patients. However, close to two-thirds of the patients still achieved LLDAS after belimumab treatment, indicating that combination therapy with belimumab and basic treatment can significantly reduce the disease activity of lupus.

On the other hand, hormones should still be used as basic drugs for treating SLE patients. Nevertheless, due to their side effects and high incidence of adverse reactions, the minimum dose of hormones should be used to control disease progression. As shown in the recommendations by the Chinese Guidelines for the Diagnosis and Treatment of Childhood−onset Systemic Lupus Erythematosus, the dosage of hormones should be adjusted to a relatively safe dose (≤5 mg/d) in the time after CR, and the lowest dose required for disease control should be applied during maintenance (30). The OBSErve study revealed that belimumab treatment can reduce the proportion of patients requiring hormone therapy and decrease the dose of hormone used in the high disease activity subgroup (22). A 13-year follow-up study also showed that the hormone consumption of SLE patients continued to decrease during the period of belimumab treatment, and the median hormone reduction was nearly 90% at 13 years (31). Similarly, a Chinese multi-center study has demonstrated that within 28 weeks of treatment, the hormone reduction in the belimumab group was more than that in the basic treatment group (29). Our study also found a significant decrease in the hormone dose during the period of belimumab treatment compared to the doses given at baseline and the previous follow-up. Among the 26 children who received belimumab, 42.3% of them stopped taking hormones and 65.4% took a reduced dose of 5 mg or less at the last follow-up. Taken together, the addition of belimumab to basic treatment is helpful to reduce the required hormone dose.

In our study, no hypersensitivity or infusion reactions occurred in the 26 children treated with belimumab. However, there was one case of fungal pneumonia, one case of upper respiratory tract infection and gastrointestinal tract infection, and one case of lower limb pain, all of which were cured after symptomatic treatment. Previous studies have found that the overall safety of belimumab is comparable to that of a placebo in adults and children with SLE (20, 32, 33), suggesting that belimumab has a good safety profile for the treatment of cSLE patients.

This study does have some limitations that should be addressed. Due to the inefficiency of a single-center real-world study, our study lacked a validation process for the obtained data. At the same time, the enrolled patients had a certain tendency, which may have led to selection bias. Also, due to a self-controlled before–after trial, the recruited patients receiving belimumab treatment all belonged to the refractory cases, meaning that the patients had active diseases and that the hormone dose could not be reduced to 5 mg or less under the current treatment. Nevertheless, the disease activity and the dose of the hormone were still significantly reduced in those patients after the addition of belimumab to the original treatment, indicating the effectiveness of treatment with belimumab.

In conclusion, through a retrospective study on 26 cSLE patients who received belimumab treatment, we revealed that the combination therapy of belimumab and conventional treatment can significantly improve laboratory indicators, decrease disease activity, and reduce hormone dosage. Through continuous follow-up, we also revealed the timing of changes in different indicators. Moreover, belimumab showed a good safety profile for the treatment of cSLE patients.

## Data availability statement

The raw data supporting the conclusions of this article will be made available by the authors, without undue reservation.

## Ethics statement

The studies involving human participants were reviewed and approved by Medical Ethics Committee of the Affiliated Hospital of Qingdao University. Written informed consent to participate in this study was provided by the participants’ legal guardian/next of kin.

## Author contributions

DW: Conceptualization; Data curation; Formal analysis; Writing - original draft. CS: Data curation; Formal analysis; Methodology. JL: Data curation; Supervision. RZ: Data curation; Supervision. GZ: Data curation. TG: Data curation. HC: Formal analysis; Investigation. SC: Data curation; Supervision. CB: Data curation; Supervision. NN: Data curation; Supervision. QZ: Conceptualization; Investigation; Project administration; Writing - review and editing. YL: Conceptualization; Investigation; Project administration; Writing - review and editing. All authors contributed to the article and approved the submitted version.
